# The Impact of Complete Revascularization in Symptomatic Severe Left Ventricular Dysfunction between Coronary Artery Bypass Graft and Percutaneous Coronary Intervention

**DOI:** 10.1155/2023/9226722

**Published:** 2023-02-28

**Authors:** Hsiu-Yu Fang, Yen-Nan Fang, Yin-Chia Chen, Jiunn-Jye Sheu, Wei-Chieh Lee

**Affiliations:** ^1^Division of Cardiology, Department of Internal Medicine, Kaohsiung Chang Gung Memorial Hospital, Chang Gung University College of Medicine, Kaohsiung, Taiwan; ^2^Division of Cardiology, Department of Internal Medicine, Jen-Ai Hospital, Taichung, Taiwan; ^3^Division of Cardiovascular Surgery, Department of Surgery, Kaohsiung Chang Gung Memorial Hospital, Chang Gung University College of Medicine, Kaohsiung, Taiwan; ^4^Institute of Clinical Medicine, College of Medicine, National Cheng Kung University, Tainan, Taiwan; ^5^Division of Cardiology, Department of Internal Medicine, Chi Mei Medical Center, Tainan, Taiwan; ^6^School of Medicine, College of Medicine, National Sun Yat-sen University, Kaohsiung, Taiwan

## Abstract

**Objective:**

The study aimed to compare the clinical outcomes between the patients receiving coronary artery bypass surgery (CABG) or percutaneous coronary intervention (PCI) for the patients with symptomatic severe left ventricular (LV) dysfunction and coronary artery disease (CAD).

**Methods:**

Between February 2007 and February 2020, a total of 745 patients who received coronary artery angiography for reduced LV ejection fraction (LVEF) < 40% and symptomatic New York Heart Association (NYHA) functional class ≥ 3 were recruited. The patients (*N* = 236) who were diagnosed with dilated cardiomyopathy or valvular heart disease without coronary artery stenosis, those with prior history of CABG or valvular surgery (*N* = 59), those who presented ST-segment elevated myocardial infarction (STEMI), those with a CAD and SYNTAX score of  ≦ 22 (*N* = 175), those who received emergent CABG for coronary perforation (*N* = 3), and those who had NYHA class ≦ 2 (*N* = 65) were excluded. Finally, 116 patients with reduced LVEF and those who had a SYNTAX score >22, who received CABG (N = 47) and PCI (N = 69), were recruited for this study.

**Results:**

There was no significant difference in the incidence values of in-hospital course and those of in-hospital mortality, acute kidney injury, and postprocedural hemodialysis. There was no significant difference in the 1-yearfollow-up of recurrent MI, revascularization, or stroke between the groups. The 1-year heart failure (HF) hospitalization rate was significantly lower in the CABG group than in all patients of the PCI group (13.2% vs. 33.3%; *p* = 0.035); however, there was no significant difference in the same variable between the CABG group and the complete revascularization subgroup (13.2% vs. 28.2%; *p* = 0.160). The revascularization index (RI) was significantly higher in the CABG group than in all patients of the PCI group or complete revascularization subgroup (0.93 ± 0.12 vs. 0.71 ± 0.25; *p* < 0.001) and (0.93 ± 0.12 vs. 0.86 ± 0.13; *p* = 0.019). The 3-year HF hospitalization rate was significantly lower in the CABG group than in all patients of the PCI group (16.2% vs. 42.2%; *p* = 0.008); however, there was no difference in the same variable between the CABG group and the complete revascularization subgroup (16.2% vs. 35.1%; *p* = 0.109).

**Conclusions:**

In patients with symptomatic (NYHA class ≥ 3) severe LV dysfunction and CAD, CABG brought less HF admission when compared to patients in the PCI group, but this did not differ when compared to the complete revascularization subgroup. Therefore, an extensive revascularization, achieved by CABG or PCI, is associated with a lower HF hospitalization rate during the 3-yearfollow-up period in such populations.

## 1. Introduction

Coronary artery disease (CAD) is one of the leading causes of severe left ventricular (LV) dysfunction and mortality in recent years [1–3]. With the improvement of healthcare in CAD, the treatment strategies are changing day after day [4]. According to the reports from the American Heart Association, both the annual rate of death due to CAD in the USA and the incidence of LV dysfunction caused by CAD were increasing [5]. This fact made us pay more attention to this combination of severe LV dysfunction and CAD.

Current American and European guidelines do not provide precise recommendations for revascularization strategies in patients with CAD and severe LV dysfunction [6, 7]. Nevertheless, the exclusion of patients with severe LV dysfunction from the clinical trial made the optimal revascularization of these patients still controversial [8–10]. Some cohort studies had concluded that coronary artery bypass surgery (CABG) was associated with a lower mortality rate and a lower major adverse cardiac event (MACE) rate than that with percutaneous coronary intervention (PCI) in patients with severe LV dysfunction CAD [11, 12]. However, the evidence was not strong enough to conclude the result of revascularization strategies. Recently, one meta-analysis showed that among the patients with severe LV dysfunction, CABG resulted in a lower mortality rate and an increased risk of stroke [13]. Surprisingly, none of these cohort studies or meta-analyses stressed on patients' clinical conditions such as hemodynamic status, New York Heart Association (NYHA) functional class, or SYNTAX score. This retrospective study aimed to explore the optimal strategy for patients with symptomatic severe LV dysfunction with NYHA class ≥3 and CAD.

## 2. Materials and Methods

The study population belonged to the HF registry of Kaohsiung Chang Gung Memorial Hospital. [14] This study was approved by the ethical committees of the institutional review board of Chang Gung Memorial Hospital (202100931B0) and the National Institution of Health of Taiwan.

### 2.1. Patients and Groups

**Figure 1:**
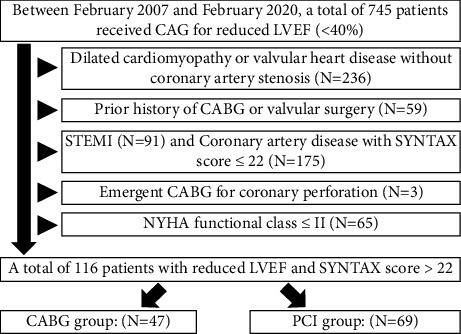
Flowchart of the patient selection process. Abbreviations: CAG: coronary angiography, LVEF: left ventricular ejection fraction, CABG: coronary artery bypass graft, STEMI: ST-segment elevation myocardial infarction, and PCI: percutaneous coronary intervention.

We retrospectively enrolled 745 patients who received coronary artery angiography for reduced LV ejection fraction (LVEF) < 40% between February 2007 and February 2020 (Figure 1). The patients (*N* = 236) who were diagnosed with dilated cardiomyopathy or valvular heart disease without coronary artery stenosis, those with prior history of CABG or valvular surgery (*N* = 59), those with ST-segment elevated myocardial infarction, those with CAD and SYNTAX scores of  ≦22 (*N* = 175), those who underwent emergency CABG for coronary perforation (*N* = 3), and those with NYHA class ≦2 (*N* = 65) were excluded. There were 116 patients with reduced LVEF and SYNTAX scores of  >22 who received CABG (*N* = 47) and PCI (*N* = 69) who were enrolled into this study. The Institutional Review Committee on Human Research at our institution approved the study protocol.

### 2.2. Definitions

Our myocardial infarction (MI) definitions followed the most recent universal definition of MI [15]. Symptomatic heart failure (HF) was defined, according to the NYHA classification, as being in a class of ≥3. Revascularization was defined as any repeat PCI in a target vessel or coronary artery bypass graft (CABG) in a target vessel for lesions with a stenosis of ≧70%. Cardiovascular (CV) mortality was defined as death due to MI, cardiac arrhythmia, or HF. All-cause mortality was defined as death from any cause. Recurrent MI was defined as acute MI occurring 1 month after the index MI. Complete revascularization was defined as the absence of any angiographic significant stenosis (>70%) in all three epicardial coronary arteries with a diameter of at least 2.5 mm after PCI.

### 2.3. Study Endpoints

The primary composite endpoints of our study were any recurrent MIs, revascularization, sudden death/ventricular arrhythmia, HF hospitalization, stroke or CV mortalities, and all-cause mortality rate during the 1- and 3-yearfollow-up periods. The secondary composite endpoints included all in-hospital events, such as in-hospital mortality, acute kidney injury, and postprocedural hemodialysis (HD).

### 2.4. Statistical Analysis

Data were expressed as the mean ± standard deviation for continuous variables or as counts and percentages for categorical variables. Continuous variables were compared using the independent sample *t*-test or the Mann–Whitney *U* test. Categorical variables were compared using the chi-square test. Univariate and multivariate cox regression analyses were performed to identify the associations relating to the 1-year CV mortality rate. Correlations between variables were expressed as hazard ratios with 95% confidence intervals. The Kaplan–Meier curves were created to illustrate the 1-year CV mortality data in each of the groups. All statistical analyses were performed using SPSS 22.0 (IBM. Corp., Armonk, NY). A *p* value of <0.05 was considered statistically significant.

## 3. Results

### 3.1. Baseline Characteristics

Table 1 presents the baseline characteristics of the study participants. There were no significant differences between the two groups in terms of demographic characteristics, including age, sex, serum creatinine level, and body mass index. There was also no significant difference in the rates of comorbidities, such as hypertension, diabetes mellitus, peripheral artery occlusive disease, chronic obstructive pulmonary disease, current smoking status, chronic kidney disease more than stage 3, and a prior MI history of >90 days between the two groups. The proportion of participants in the CABG group with the end-stage renal disease was significantly higher than that in all patients in the PCI group (21.3% vs. 7.2%; *p*=0.046); however, the difference in the same parameter between the CABG group and the complete revascularization subgroup was not statistically significant (21.3% vs. 11.9%; *p*=0.270). There were also significantly more patients with a prior history of PCI in the CABG group than in all patients in the PCI group (55.3% vs. 13.0%; *p* < 0.001) and the complete revascularization subgroup (55.3% vs. 11.9%; *p* < 0.001). The major clinical presentation was acute coronary syndrome (89.4% vs. 76.8% vs. 85.7%) other than stable angina or HF.

The surgical risk and coronary complexity parameters such as the New EuroScore II were higher in the CABG group than in all patients of the PCI group or the complete revascularization subgroup (9.2 ± 7.2 vs. 3.6 ± 2.0; *p* < 0.001) or (9.2 ± 7.2 vs. 3.5 ± 1.7; *p* < 0.001). The SYNTAX score was also higher in the CABG group than in all patients of the PCI group or the complete revascularization subgroup (40.4 ± 10.5 vs. 35.9 ± 8.2; *p*=0.010) or (40.4 ± 10.5 vs. 35.4 ± 8.0; *p*=0.014). Further analysis revealed no significant difference in coronary complexity parameters such as SYNTAX score > 33, left main disease, multivessel disease, two-vessel disease, three-vessel disease, or chronic total occlusion (CTO) between the groups.

The complete revascularization parameter was assessed by the British Cardiovascular Intervention Society's myocardial jeopardy score (BCIS-JS) [16, 17] before and after PCI. The revascularization index was calculated by (preBCIS-JS-postBCIS-JS)/(preBCIS-JS). The BCIS-JS before the procedure was also significantly higher in the CABG group than in all patients of the PCI group or the complete revascularization subgroup (11.8 ± 0.6 vs. 10.6 ± 1.7; *p* < 0.001) and (11.8 ± 0.6 vs. 10.4 ± 3.0; *p* < 0.001). The BCIS-JS after the procedure was also significantly higher in the CABG group than in all patients of the PCI group (0.9 ± 0.4 vs. 3.1 ± 2.8; *p* < 0.001), but no significant difference was observed in the CABG group and the complete revascularization subgroup (0.9 ± 0.4 vs. 1.4 ± 1.3; *p*=0.064). Both the revascularization index (RI) was higher than 0.67 in all groups but still significantly higher in the CABG group than in all patients of the PCI group or the complete revascularization subgroup (0.93 ± 0.12 vs. 0.71 ± 0.25; *p* < 0.001) and (0.93 ± 0.12 vs. 0.86 ± 0.13; *p*=0.019).

There was also no significant difference in the need for mechanical support before, during, and after the procedure (34.0% vs. 36.2%, *p*=0.845; 34.0% vs. 31.0%, *p*=0.823). Most of the timing of mechanical support was before the procedure and maintained 24 hours after the procedure. The type of mechanical support such as extracorporeal membrane oxygenation (ECMO) (0% vs. 4.3%, *p*=0.271; 0% vs. 7.1%, *p*=0.101) and intraaortic balloon pump (IABP) (34.0% vs. 36.2%, *p*=0.845, 34.0% vs. 31.0%, *p*=0.823) did not show differences in both the groups. There was no Impella used in the study groups.

There was no significant difference in the rate of use of ACEI/ARB/ARNi and MRA between the groups. However, the rate of use of beta-blockers was significantly lower in the CABG group than in all patients of the PCI group (62.2% vs. 85.3%; *p*=0.007) or the complete revascularization subgroup (62.2% vs. 87.8%; *p*=0.007). Also, the rate of use of furosemide was also significantly lower in the CABG group than in all patients of the PCI group (31.9% vs. 59.4%; *p*=0.005) or the complete revascularization subgroup (31.9% vs. 50.0%; *p*=0.090). There was no significant difference in the mean follow-up duration between the groups.

### 3.2. Echocardiographic Parameters

Table 2 demonstrates the echocardiography parameters at the baseline and follow-up. There were no significant differences in baseline echocardiography parameters such as LA dimension, LVEF, LVEF < 30%, LVEDV, LVESV, AR grade > 2, MR grade > 2, TR grade > 2, and TRPG between the CABG group and all patients of the PCI group. Among the follow-up echocardiography parameters, there were no significant differences in LA dimension, LVEF, LVEF > 50%, LVEF > 40%, LVEDV, LVESV, AR grade > 2, MR grade > 2, TR grade > 2, and TRPG between the CABG group and all patients in the PCI group. There were also no significant differences in a reduction in LVEDV of > 10% or a reduction in LVESV of > 10% between the CABG group and in all patients of the PCI group. There were no significant differences in the improvements in LVEF or mean LVEF > 10% between the CABG group and in all patients of the PCI group and the complete revascularization subgroup (14.7 ± 15.5 vs. 15.8 ± 17.3 vs. 18.9 ± 16.6) and (62.8% vs. 64.3% vs. 69.7%).

### 3.3. Clinical Outcomes

Table 3 illustrates the long-term clinical outcomes of groups. There were no significant differences in the incidences of in-hospital course and in-hospital mortality, acute kidney injury (AKI), and postprocedural HD. There were no differences in primary composite endpoints at 1-year or 3-yearfollow-up comparing CABG vs. all patients in the PCI group and the complete revascularization subgroup (29.8% vs. 42.0%, *p*=0.240; 29.8% vs. 40.5%, *p*=0.374) (42.6% vs. 53.6%, *p*=0.262; 42.6% vs. 52.4%, *p*=0.399). There was no statistically significant difference in the 1-yearfollow-up between the groups of recurrent MI, revascularization, or stroke, and they were similar; however, the 1-year HF hospitalization rate was significantly lower in the CABG group than in all patients of the PCI group (13.2% vs. 33.3%; *p*=0.035); however, the difference in the same parameter between the CABG group and the complete revascularization subgroup was not statistically significant (13.2% vs. 28.2%; *p*=0.160). The incidences of sudden death/ventricular arrhythmia, CV mortality, and all-cause mortality were higher in the CABG group; however, the differences were not statistically significant.

There was no statistically significant difference in the 3-yearfollow-up between the recurrent MI, revascularization, and stroke groups. The 3-year HF hospitalization rate was significantly lower in the CABG group than in all patients of the PCI group (16.2% vs. 42.2%; *p*=0.008); however, the difference in the same parameter between the CABG group and the complete revascularization subgroup was not statistically significant (16.2% vs. 35.1%; *p*=0.109). The incidences of sudden death/ventricular arrhythmia, CV mortality, and all-cause mortality were higher in the CABG group, although the differences were not statistically significant.

### 3.4. Kaplan–Meier Curves Comparing the 1- and 3-YearAll-Cause Mortality Rates between CABG and PCI

**Figure 2:**
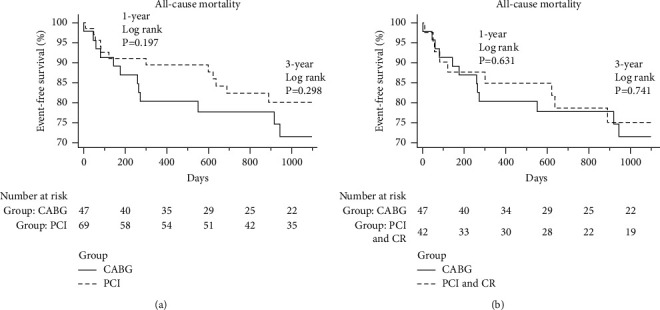
Kaplan–Meier curves of 1- and 3-yearall-cause mortality rates between the groups. (a) The Kaplan–Meier curve of the 1- and 3-yearall-cause mortality rates between CABG and in all patients in the PCI group and (b) the Kaplan–Meier curve of the 1- and 3-yearall-cause mortality rates between the CABG and the complete revascularization subgroup.

Figure 2 shows the Kaplan–Meier curve illustrating the differences in the 1- and 3-yearall-cause mortality rates between the groups. There was no significant difference in the 1-yearall-cause mortality rate between the CABG group and in all patients of the PCI group (*p*=0.197) and between the CABG group and the complete revascularization subgroup (*p*=0.631). There was no significant difference in the 3-yearall-cause mortality rate between the CABG group and in all patients of the PCI group (*p*=0.298) and between the CABG group and the complete revascularization subgroup (*p*=0.741).

### 3.5. Kaplan–Meier Curves Comparing 1- and 3-Year HF Hospitalization Rates between CABG and PCI

**Figure 3:**
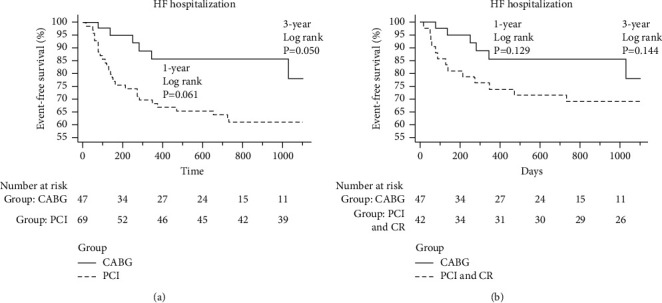
Kaplan–Meier curves of the 1- and 3-year HF hospitalization rates between the groups. (a) The Kaplan–Meier curve of the 1- and 3-year HF hospitalization rates between CABG and all patients in the PCI group and (b) the Kaplan–Meier curve of the 1- and 3-year HF hospitalization rates between the CABG and the complete revascularization subgroup.

Figure 3 shows the Kaplan–Meier curve illustrating the difference in the 1- and 3-year HF hospitalization rates between the groups. There was some trend lower but without a statistically significant difference in the 1-year HF hospitalization rate between the CABG group and in all patients of the PCI group (*p*=0.061) or between the CABG group and the complete revascularization subgroup (*p*=0.129). There was also some trend lower but without a statistically significant difference in the 3-year HF hospitalization rate between the CABG group and in all patients of the PCI group (*p*=0.050) and between the CABG group and the complete revascularization subgroup (*p*=0.144).

## 4. Discussion

The most common cause of severe LV dysfunction is coronary artery disease. Despite numerous studies made, atherogenesis and its progression leading to endothelial injury remain multifactorial which include smoking, hypertensive trigger, and oxidative stress. One of the most definite progressions of atherosclerosis is coronary artery calcification. Coronary artery calcification has been used for staging coronary atherosclerosis. The mechanism of coronary artery calcification may lie in the release of apoptotic/necrotic bodies, the release of matrix vesicles, and the differentiation of pericytes during the plaque calcification process [18]. Survivors after adequate treatment of acute coronary syndrome and myocardial infarction may develop severe LV dysfunction due to irreversible myocyte loss with scar formation, hibernating myocardium, or adverse cardiac remodeling [19].

The meta-analysis results of patients with severe LV dysfunction and CAD who were treated with CABG had a higher risk of stroke during the short-termfollow-up; however, they had a lower risk of death, MI, and repeat revascularization than those treated with PCI during the long-termfollow-up [13]. Randomized controlled trials on revascularization strategies in patients with severe LV dysfunction CAD are rare, except for the Heart Failure Revascularization Trial [20] and Surgical Treatment for Ischemic Heart Failure Extension Study [21]. Both of these randomized controlled trials were not set up by comparing CABG and PCI in patients with severe LV dysfunction. Conversely, most randomized trials only compared CABG with PCI in patients whose LV function was not severely depressed.

The NYHA classification has served as a fundamental tool for the risk stratification of HF and for determining the clinical trial eligibility and candidacy for devices and drugs. Recently, one systemic review showed that the NYHA system poorly discriminated against patients with HF across the spectrum of functional impairment [22]. However, other studies showed that the NYHA classification, along with other comorbidities, still might be helpful in identifying a subgroup of implantable cardioverter defibrillator carriers with poor prognoses and a higher risk of CV death [23]. To the best of our knowledge, there is no existing study that compares CABG and PCI in patients with symptomatic severe LV dysfunction CAD.

Current evidence from randomized trials suggests that the risk of mortality and MI does not differ significantly among various revascularization strategies for multivessel CAD. A meta-analysis showed that complete revascularization of multivessel CAD was associated with a reduction in the rate of MACE due to the reduction in emergency revascularization [24]. However, the importance of complete revascularization had been addressed only to reduce repeat revascularization and subsequent major adverse cardiac and cerebral events in patients with LV dysfunction but not to reduce the long-term mortality rate [25]. One of the most important reasons that complete revascularization cannot achieve long-term clinical outcome improvement is the variety of complete revascularization definitions. In the COMPLETE trial [26], the definition of complete revascularization was on the completeness of nonculprit lesion PCI which is defined as the vessel which is larger than 2.5 mm with angiographic more than 70% stenosis or with 50 to 69% stenosis but a fractional flow reserve (FFR) of less than 0.80. The DANAMI-3-PRIMULTI trial [27] also defined complete revascularization as revascularization of all coronary lesions which are greater than 50% in diameter stenosis and larger than 2.0 mm in diameter which was confirmed by FFR. Another reason that complete revascularization cannot achieve long-term clinical outcome improvement is because the extensive revascularization is not well defined. The British Cardiovascular Intervention Society's myocardial jeopardy score (BCIS-JS) was developed to define the completeness of coronary revascularization [16]. Revascularization index (RI) was defined as (pre-BCIS-JS-postBCIS-JS)/(pre-BCIS-JS) which showed that RI < 0.67 is associated with a higher mortality rate. In our study, the RIs were significantly lower in all patients of the PCI group and the complete revascularization subgroup compared with the CABG group suggesting that the degree of extensive revascularization is still different. In our study, the long-term mortality rate did not differ significantly in CABG, all patients in the PCI group, or the complete revascularization subgroup, and even in patients with symptomatic (NYHA class more than 3) and severe LV dysfunction who had less HF admission due to the differences in complete revascularization.

Elective insertion of the hemodynamic support device in complex high-risk indicated patients (CHIP) may be reasonable as an adjunct to PCI or CABG which was a class IIb recommendation according to guidelines [28]. BCIS-1 study showed that in patients with severe ischemic cardiomyopathy treated with PCI, elective IABP use during PCI was associated with a 34% relative reduction in all-cause mortality compared with unsupported PCI [29]. The evidence of prophylactic ECMO in CHIP PCI was insufficient which also showed benefits in case series results [30, 31]. PROTECT II study showed that in 452 symptomatic patients with complex three-vessel coronary artery disease or unprotected left main coronary artery disease and severe depressed left ventricular function underwent nonemergent PCI, although the 30-day incidence of major adverse events was not different between IABP and Impella 2.5, but improved outcomes were observed for Impella 2.5 group at 90 days with significant maintenance of mean arterial pressure during PCI [32, 33]. Roma-Verona registry also demonstrated that the Impella-protected PCI could achieve more complete revascularization with a higher revascularization index with significant improvement in the left ventricular ejection fraction and survival [34]. The Impella-supported intervention also showed its benefit not only in CHIP PCI but also in cardiogenic shock patients in the IMP-IT registry [35]. This evidence provides us more confidence in extensive revascularization under mechanical support such as IABP, ECMO, or Impella. However, bleeding complications under mechanical support are common. One of the most leading causes of bleeding complications was the cleavage of von Willebrand factor (VWF) when using ECMO or left ventricular assist devices (LVADs). The basic VWF monomer contains 2050 amino acids making the protein sensitive to changes in fluid flow and shear stress. LVADs have been associated with an increased bleeding risk, commonly attributed to hydrodynamic changes and shear stress induced by continuous flow across the devices, which augmented VWF cleavage also seen in severe LV dysfunction patients [36]. These made one-stage surgery such as CABG instead of prolonging the use of mechanical support devices more suitable for clinical decision-making [37].

### 4.1. Limitations

Our study had several limitations. First, we enrolled patients from 2007 to 2020 due to the limited number of patients, which represented selection bias. Second, the PCI and CABG were all operator-dependent procedures that influenced the true clinical outcome. Third, there was no analysis between drug-eluting stents or bare-metal stents in the PCI group.

## 5. Conclusions

In patients with symptomatic (NYHA class ≥ 3) severe LV dysfunction and CAD, CABG brought less HF admission when compared to patients in the PCI group but did not differ when compared to the complete revascularization subgroup. Therefore, extensive revascularization, achieved by CABG or PCI, is associated with a lower HF hospitalization rate during a 3-yearfollow-up period in such populations.

## Figures and Tables

**Table 1:** Baseline characteristics of the study patients.

	CABG group	PCI group		*p* value	*p* value
		All patients	CR	CABG vs. All PCI	CABG vs. CR
Number	47	69	42		
General demographics					
Age (years)	63 ± 9.1	65 ± 10.6	63 ± 9.7	0.285	0.880
Male sex (%)	41 (87.2)	56 (81.2)	31 (73.8)	0.451	0.176
BMI (kg/m^2^)	24.9 ± 3.6	24.7 ± 4.5	24.2 ± 3.9	0.817	0.340
Comorbidities					
Hypertension (%)	36 (76.6)	45 (65.2)	25 (59.5)	0.221	0.110
Diabetes mellitus (%)	28 (59.6)	46 (66.7)	29 (69.0)	0.440	0.384
PAOD (%)	1 (2.1)	6 (8.7)	2 (4.8)	0.238	0.600
COPD (%)	2 (4.3)	2 (2.9)	1 (2.4)	1.000	1.000
ESRD (%)	10 (21.3)	5 (7.2)	5 (11.9)	0.046	0.270
CKD stage ≥ 3 (%)	27 (57.4)	32 (46.4)	22 (52.4)	0.262	0.674
Smoking (%)	25 (53.2)	34 (49.3)	19 (45.2)	0.709	0.526
Prior PCI history (%)	26 (55.3)	9 (13.0)	5 (11.9)	<0.001	<0.001
Prior MI < 90 days (%)	21 (44.7)	28 (40.6)	20 (47.6)	0.704	0.833
Clinical presentation				0.093	0.750
Acute coronary syndrome (%)	42 (89.4)	53 (76.8)	36 (85.7)		
Stable angina/HF (%)	5 (10.6)	16 (23.2)	6 (14.3)		
Laboratory examination					
Creatinine (exclude ESRD) (mg/dL)	1.71 ± 0.70	1.55 ± 0.32	1.65 ± 0.61	0.596	0.890
New EuroSCORE II	9.2 ± 7.2	3.6 ± 2.0	3.5 ± 1.7	<0.001	<0.001
Coronary complexity					
SYNTAX score	40.4 ± 10.5	35.9 ± 8.2	35.4 ± 8.0	0.010	0.014
SYNTAX score >33 (%)	35 (74.5)	41 (59.4)	26 (61.9)	0.113	0.255
Left main (%)	16 (34.0)	19 (27.5)	13 (31.0)	0.538	0.823
MVD				0.818	0.615
2-V-D (%)	9 (19.1)	15 (21.7)	10 (23.8)		
3-V-D (%)	38 (80.9)	54 (78.3)	32 (76.2)		
CTO (%)	29 (61.7)	42 (60.9)	21 (50.0)	1.000	0.292
Complete revascularization parameter					
Pre-BCIS-JS	11.8 ± 0.6	10.6 ± 1.7	10.4 ± 3.0	<0.001	<0.001
Post-BCIS-JS	0.9 ± 0.4	3.1 ± 2.8	1.4 ± 1.3	<0.001	0.064
RI	0.93 ± 0.12	0.71 ± 0.25	0.86 ± 0.13	<0.001	0.019
Requirement of mechanical support before, during, and after (%)	16 (34.0)	25 (36.2)	13 (31.0)	0.845	0.823
ECMO	0 (0)	3 (4.3)	3 (7.1)	0.271	0.101
IABP	16 (34.0)	25 (36.2)	13 (31.0)	0.845	0.823
Medication					
ACEI/ARB/ARNi use (%)	32 (71.1)	53 (77.9)	32 (78.0)	0.505	0.621
*β*-blocker use (%)	28 (62.2)	58 (85.3)	36 (87.8)	0.007	0.007
MRA (%)	23 (51.1)	20 (29.4)	15 (36.6)	0.029	0.198
Furosemide (%)	15 (31.9)	41 (59.4)	21 (50.0)	0.005	0.090
F/U duration (days)	1192 ± 1115	1122 ± 794	1094 ± 891	0.693	0.651

Data are expressed as the mean ± standard deviation or as number (percentage). Abbreviations: CABG, coronary artery bypass graft; PCI, percutaneous coronary intervention; CR, complete revascularization; BMI, body mass index; kg, kilogram; m, meter; PAOD, peripheral arterial occlusive disease; COPD, chronic obstructive pulmonary disease; ESRD, end-stage renal disease; CKD, chronic kidney disease; MI, myocardial infarction; HF, heart failure; MVD, multiple vessel disease: CTO, chronic total occlusion; BCIS-JS, British Cardiovascular Intervention Society myocardial jeopardy score; RI, revascularization index (preBCIS-JS-postBCIS-JS)/(preBCIS-JS); ECMO, extracorporeal membrane oxygenation, IABP, intraaortic balloon pump; ACEI, angiotensin-converting enzyme inhibitor; ARB, angiotensin receptor blocker; ARNi, angiotensin receptor-neprilysin inhibitor; MRA, mineralocorticoid receptor antagonist; F/U, follow-up.

**Table 2:** The parameters of the baseline and follow-up echocardiography.

	CABG group	PCI group		*p* value	*p* value
		All patients	CR	CABG vs. All PCI	CABG vs. CR
Number	47	69	42		
Baseline					
LA dimension (mm)	40.1 ± 6.3	41.8 ± 6.4	41.6 ± 5.7	0.172	0.243
LVEF (%)	30.4 ± 6.7	31.2 ± 7.0	30.2 ± 6.8	0.569	0.913
<30% (%)	23 (48.9)	25 (36.2)	17 (40.5)	0.185	0.523
LVEDV (ml)	178.1 ± 39.0	178.7 ± 56.8	182.6 ± 61.6	0.948	0.676
LVESV (ml)	124.2 ± 35.2	123.3 ± 46.1	127.8 ± 50.2	0.918	0.689
AR grade > 2 (%)	1 (2.1)	9 (13.0)	5 (11.9)	0.047	0.096
MR grade > 2 (%)	18 (38.3)	27 (39.1)	17 (40.5)	1.000	1.000
TR grade > 2 (%)	7 (14.9)	14 (20.3)	7 (16.7)	0.624	1.000
TRPG (mmHg)	27.6 ± 15.5	27.3 ± 13.9	26.3 ± 14.9	0.920	0.712
Follow-up					
LA dimension (mm)	40.9 ± 7.3	40.0 ± 7.4	38.6 ± 7.7	0.510	0.182
LVEF (%)	44.7 ± 14.4	46.7 ± 15.4	48.9 ± 18.9	0.530	0.229
>50% (%)	19 (44.2)	25 (44.6)	16 (48.5)	1.000	0.817
>40% (%)	25 (58.1)	36 (64.3)	23 (69.7)	0.540	0.344
LVEDV (ml)	168.2 ± 66.4	166.0 ± 62.5	164.3 ± 63.4	0.864	0.797
LVESV (ml)	98.5 ± 59.3	93.9 ± 52.1	90.5 ± 53.5	0.682	0.546
AR grade > 2 (%)	0 (0)	4 (7.1)	1 (3.0)	0.130	0.434
MR grade > 2 (%)	7 (16.3)	9 (16.1)	6 (18.2)	1.000	1.000
TR grade > 2 (%)	5 (11.6)	4 (7.1)	2 (6.1)	0.496	0.692
TRPG (mmHg)	25.3 ± 14.5	24.3 ± 17.2	20.6 ± 14.2	0.763	0.186
The change of left ventricular volume between baseline and follow-up					
Reducing LVEDV > 10% (%)	20 (46.5)	28 (50.0)	17 (51.5)	0.840	0.817
Reducing LVESV > 10% (%)	29 (67.4)	32 (57.1)	19 (57.6)	0.405	0.473
The change of LVEF between baseline and follow-up	14.7 ± 15.5	15.8 ± 17.3	18.9 ± 16.6	0.749	0.263
Improving mean LVEF > 10% (%)	27 (62.8)	36 (64.3)	23 (69.7)	1.000	0.628

Data are expressed as the mean ± standard deviation or as number (percentage). Abbreviations: CABG, coronary artery bypass graft; PCI, percutaneous coronary intervention; CR, complete revascularization; LA, left atrium; LVEF, left ventricular ejection fraction; LVEDV, left ventricular end-diastolic volume; LVESV, left ventricular end-diastolic volume; AR, aortic regurgitation; MR, mitral regurgitation; TR, tricuspid regurgitation: TRPG, tricuspid regurgitation pressure gradient.

**Table 3:** Clinical outcomes of the study patients.

	CABG group	PCI group		*p* value	*p* value
		All patients	CR	CABG vs. All PCI	CABG vs. CR
Number	47	69	42		
In-hospital course					
In-hospital mortality (%)	2 (4.3)	1 (1.4)	1 (2.4)	0.565	1.000
AKI (%)	3 (8.1)	14 (21.9)	7 (18.9)	0.099	0.308
Postprocedural HD (%)	2 (5.4)	4 (6.3)	3 (8.1)	1.000	1.000
One-yearfollow-up duration					
Primary endpoints (%)	14 (29.8)	29 (42.0)	17 (40.5)	0.240	0.374
Recurrent MI (%)	2 (5.0)	5 (7.9)	2 (5.4)	0.703	1.000
Revascularization (%)	2 (5.6)	6 (10.9)	2 (6.7)	0.471	1.000
Sudden death/Ventricular arrhythmia (%)	5 (11.6)	2 (3.1)	1 (2.7)	0.114	0.209
HF hospitalization (%)	5 (13.2)	22 (33.3)	11 (28.2)	0.035	0.160
Stroke (%)	0 (0)	0 (0)	0 (0)	—	—
CV mortality (%)	8 (17.4)	5 (7.5)	4 (10.0)	0.136	0.367
All-cause mortality (%)	9 (19.1)	7 (10.1)	6 (14.3)	0.182	0.583
Three-yearfollow-up duration					
Primary composite endpoints (%)	20 (42.6)	37 (53.6)	22 (52.4)	0.262	0.399
Recurrent MI (%)	4 (10.8)	5 (8.6)	2 (5.9)	0.732	0.675
Revascularization (%)	4 (19.0)	9 (24.3)	4 (21.1)	0.751	1.000
Sudden death/Ventricular arrhythmia (%)	8 (18.6)	4 (6.6)	2 (5.7)	0.069	0.171
HF hospitalization (%)	6 (16.2)	27 (42.2)	13 (35.1)	0.008	0.109
Stroke (%)	1 (2.1)	0 (0)	0 (0)	0.405	1.000
CV mortality (%)	11 (23.9)	7 (10.9)	6 (15.4)	0.115	0.418
All-cause mortality (%)	12 (25.5)	12 (17.4)	9 (21.4)	0.352	0.803

Data are expressed as the mean ± standard deviation or as number (percentage). Abbreviations: CABG, coronary artery bypass graft; PCI, percutaneous coronary intervention; CR, complete revascularization; AKI, acute kidney injury; HD, hemodialysis; MI, myocardial infarction; HF, heart failure; CV, cardiovascular.

## Data Availability

The data used to support the findings of the study can be obtained from the corresponding author upon request.
